# Yogurt as a modulator of gut and beyond gut: Mechanisms, health effects, and clinical translation

**DOI:** 10.3934/microbiol.2026017

**Published:** 2026-06-26

**Authors:** Daiyu Yang, Hongwen Zhao, Kun He, Wangyang Chen, Hemiao Xu, Shuai Li, Qiming Xiao, Jinshui Yang, Dong Wu

**Affiliations:** 1 State Key Laboratory of Complex Severe and Rare Diseases, Department of Gastroenterology, Peking Union Medical College Hospital, Chinese Academy of Medical Sciences and Peking Union Medical College, Beijing 100730, China; 2 Chinese Academy of Medical Sciences and Peking Union Medical College, Beijing 100730, China; 3 State Key Laboratory of Animal Biotech Breeding, College of Biological Sciences, China Agricultural University, Beijing, China; 4 Department of Gastroenterology, The People's Hospital of Xizang Autonomous Region, Lhasa 850000, China

**Keywords:** yogurt, gut microbiota, probiotics, prebiotics, health effects, microbiome modulation, precision nutrition, clinical translation

## Abstract

Yogurt, a fermented dairy food, has been increasingly recognized for its potential to modulate gut microbiota and promote host health. Accumulating evidence suggests that yogurt consumption influences gut microbial composition, diversity, and functional activity. In this narrative review, we synthesized the findings on yogurt-related effects on the gut microbiota, intestinal barrier, microbial metabolites, immune responses, and selected extra-intestinal outcomes. We distinguished traditional yogurt, probiotic yogurt, synbiotic yogurt, fortified yogurt, and non-dairy or regional yogurt-like fermented products, and then organized proposed mechanisms into a hierarchical framework that separated direct yogurt-derived inputs, including starter cultures, added probiotic strains, fermentation-derived compounds, and dairy matrix components, from resident microbiota-mediated secondary metabolites and host downstream responses. Importantly, limitations and controversies, such as variability in yogurt formulations, strain-specific effects, and inter-individual responses, were critically evaluated. Finally, we highlighted future research directions that emphasize standardized study designs, defined endpoints, long-term randomized controlled trials, and integrative multi-omics approaches to support the development of personalized dietary strategies. Together, this review provides a structured framework for understanding the complex interactions between yogurt, gut microbiota, and host physiology, while outlining key steps needed to translate evidence into actionable nutritional recommendations.


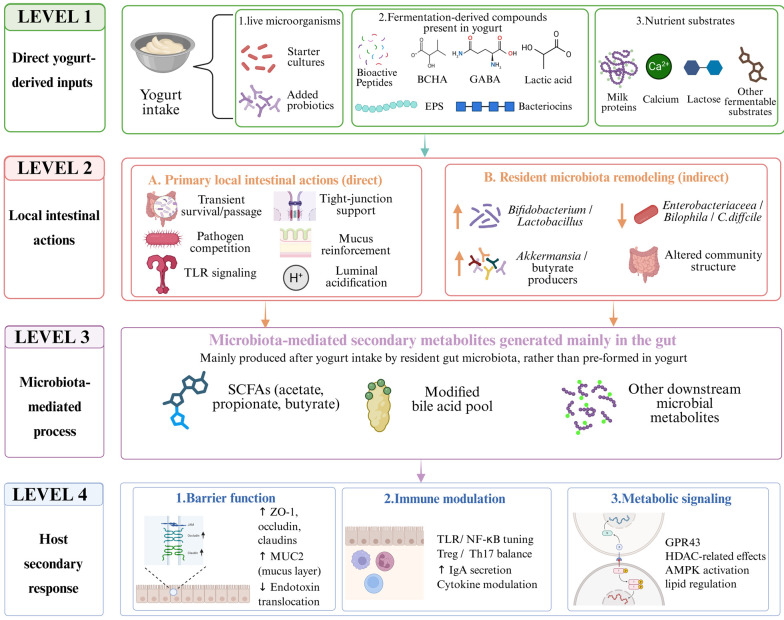


## 1. Introduction

The human gastrointestinal tract is home to a complex ecosystem of trillions of microorganisms, collectively known as the gut microbiota. This microbial community plays a pivotal role in maintaining host homeostasis by regulating metabolic pathways, fortifying the intestinal barrier, and modulating the immune system [1]. Disruptions in the composition and functional balance of the gut microbiota, dysbiosis, are increasingly implicated in the pathogenesis of a wide spectrum of disorders [2]. Consequently, dietary strategies that influence the gut microbiome have attracted interest as potential approaches for supporting health and disease-risk management.

Diet represents one of the most potent modulators of gut microbial composition and activity. Within this context, fermented foods, particularly yogurt, have emerged as prominent candidates for dietary interventions targeting gut health [3],[4]. Yogurt, a product of milk fermentation by specific bacterial cultures, has been consumed for centuries and is recognized not only as a nutrient dense food but also as a functional food with potential health promoting properties [5]. Its significance lies in its unique matrix, which delivers viable microorganisms, fermentation derived bioactive metabolites, and essential nutrients like calcium and high quality protein [6].

Importantly, the term “yogurt” in this review encompasses several categories of fermented dairy products, including traditional yogurt produced with traditional starter cultures (*Streptococcus thermophilus* and *Lactobacillus delbrueckii* subsp. *bulgaricus*), probiotic yogurt (supplemented with documented probiotic strains such as *Bifidobacterium* spp.), synbiotic yogurt (combined probiotics and prebiotics), and fortified yogurt products (containing added polyphenols, vitamins, or other bioactives). Where relevant, non-traditional yogurt-like fermented systems (e.g., soy yogurt or regional fermented milk products such as Tibetan yak yogurt) are discussed separately for mechanistic comparison rather than being considered equivalent to conventional yogurt. This distinction is critical because the microbial composition, metabolite profiles, and functional effects vary substantially across these categories, and treating them as homogeneous is a major limitation in other reviews.

Epidemiological evidence consistently links yogurt consumption to improved health outcomes. Observational studies suggest associations between regular yogurt intake and reduced risks of a wide range of disorders [7],[8]. Clinical trials and mechanistic studies further support these observations, suggesting that yogurt consumption can transiently increase the abundance of its constituent bacteria within the gut, enhance microbial diversity, stimulate short chain fatty acids (SCFAs) production, modulate immune responses, strengthen intestinal barrier function, and reduce systemic inflammation [9]–[11]. However, as discussed throughout this review, many of these findings derive from preclinical models or short-term interventions, and causality has not been firmly established for most outcomes.

Despite its long history of consumption and documented health benefits, the mechanisms through which yogurt-derived microbes and metabolites interact with the host's endogenous microbiota remain incompletely understood. Furthermore, the high degree of inter-individual variability in response to yogurt intervention, driven by baseline microbial diversity and host genetics, poses a significant challenge for standardized clinical application. In this review, we provide a comprehensive synthesis of how yogurt shapes microbial composition and diversity, discuss its multifaceted roles in metabolic and immune regulation across health states, and evaluate barriers to translating yogurt-related evidence into standardized, evidence-informed dietary strategies.

## 2. Methods

This review was conducted as a narrative review aimed at summarizing evidence regarding the interactions between yogurt, gut microbiota, and host health. Literature searches were performed using PubMed, and Web of Science for studies published from January 1, 2000 to January 1, 2026. Detailed search strategies are listed in the Supplementary File. Preclinical and clinical studies were considered, including *in vitro* studies, animal experiments, observational human studies, randomized controlled trials, and relevant reviews. Priority was given to peer-reviewed English-language articles focusing on yogurt or yogurt-related fermented dairy products and their microbiota-associated effects. Studies involving non-dairy fermented products or probiotic supplements unrelated to yogurt matrices were included only when they provided mechanistic insights relevant to yogurt-associated microbial or metabolic pathways. Articles lacking sufficient methodological information or not directly related to gut microbiota modulation were excluded. Given the heterogeneity of yogurt formulations, microbial strains, and study designs, we aim to provide a qualitative synthesis rather than a formal meta-analysis.

## 3. Classification, microbial composition, and bioactive metabolites of yogurt

**Figure 1.**
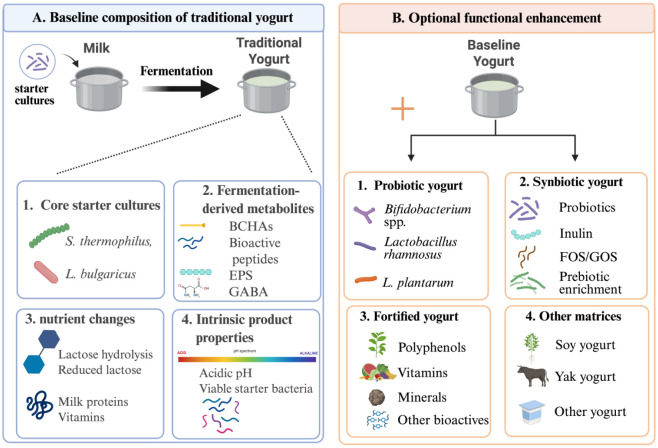
Microbial composition and bioactive metabolites of different yogurt categories. The figure illustrates the core components of traditional and functional yogurts, highlighting (1) starter cultures used in traditional yogurt (*Streptococcus thermophilus* and *Lactobacillus delbrueckii* subsp. *bulgaricus*) [13], (2) additional probiotic strains commonly incorporated into probiotic yogurt (e.g., *Bifidobacterium* spp., *Lactobacillus acidophilus*) [14], and (3) bioactive fermentation-derived metabolites, including bioactive peptides, and exopolysaccharides [15]. Optional fortification elements in synbiotic or fortified yogurts are depicted separately to indicate their additional, non-core contributions to microbial activity or host physiology. Non-dairy yogurt analogs, such as soy-based products, are distinguished due to differences in microbial composition and metabolic profiles.

Yogurt represents a diverse category of fermented foods whose microbial composition and bioactive metabolites vary substantially depending on production methods, starter cultures, and added functional ingredients. These microbial and metabolic differences underlie the distinct physiological and gut-modulating properties attributed to different yogurt types. Therefore, clear reporting of bacterial strains, viable counts, matrix composition, and added nutrients is essential for improving comparability across studies and for interpreting potential gut-related effects. This helps make sure that research studies on yogurt are consistent and accurate, enabling better understanding of how yogurt can improve gut health and overall well-being [12]. We provide an integrated overview of the core microbial communities and fermentation-derived compounds present in yogurt, and then systematically describe how these features differ among traditional yogurt, probiotic yogurt, synbiotic yogurt, fortified yogurt, and non-dairy yogurt analogs. Figure 1 provides a detailed representation of the major microbial and biochemical components of different yogurts. The figure highlights the major starter cultures, including *Lactobacillus delbrueckii* subsp. *bulgaricus* and *Streptococcus thermophilus*, as well as additional probiotic strains commonly incorporated into yogurt. In parallel, key fermentation-derived metabolites, such as bioactive peptides, and exopolysaccharides, are presented. By illustrating the coexistence and interactions of these components, Figure 1 emphasizes yogurt as a biologically active system and provide a mechanistic basis for its capacity to modulate gut microbiota composition and metabolic function.

### 3.1. Traditional yogurt

Traditional yogurt fermentation is produced using two starter cultures, *S. thermophilus* and *L. delbrueckii* subsp. *bulgaricus*, whose co-growth drives rapid acidification, proteolysis of caseins, and gel formation. These starters determine the baseline sensory and rheological properties of yogurt and contribute to early postbiotic formation [16]. Traditional yogurt inherently contains live bacteria and fermentation-derived metabolites that may transiently interact with the resident gut microbiota [13].

Although the starter strains typically do not permanently colonize the gut, they can influence the gut environment by producing lactic acid and bioactive peptides that may influence the local luminal environment and nutrient digestion. Studies have shown that these core cultures may stimulate the growth of resident commensals, foster lactic acid production in the colon, and improve lactose digestibility via residual lactase activity [17].

### 3.2. Probiotic yogurt

Probiotic yogurt is distinguished by the addition of specific probiotic strains beyond the traditional starter cultures. These may include *Bifidobacterium longum*, *B. bifidum*, *Lactobacillus acidophilus*, and others [14]. The inclusion of additional probiotic strains is intended to increase the diversity of live microbial inputs and enhance functional attributes compared with traditional yogurt. Probiotic strains must be present in sufficient viable counts at the time of consumption to be considered probiotic, and their persistence and activity in the gastrointestinal tract depend on strain characteristics, matrix interactions, and host factors.

While traditional yogurt cultures may transiently influence gut microbial communities, added probiotic strains are hypothesized to provide more specific mechanistic effects on immune modulation, barrier integrity, and metabolic functions. Among the most commonly incorporated probiotics are *Bifidobacterium animalis* ssp. *lactis* BB-12® and *B. longum*, which exhibit intrinsic acid tolerance, bile-salt hydrolase activity, and mannose specific adhesins that may facilitate transient mucosal association [18]–[20]. Additional bacteria, *Propionibacterium freudenreichii*, synthesizing propionate and cobalamin, and *Lactococcus lactis*, a nisin producer, extend the metabolic function toward vitamin enrichment and targeted antimicrobial activity [21]–[23]. However, the extent to which these strains achieve sustained colonization or produce durable health effects remains incompletely understood and likely varies among individuals.

### 3.3. Synbiotic yogurt

Synbiotic yogurt integrates probiotics with prebiotic substrates or related functional components intended to stimulate beneficial microbial activity in the gut. In most synbiotic yogurt, prebiotic fibers such as inulin, fructooligosaccharides (FOS), and galactooligosaccharides (GOS) are incorporated into the yogurt matrix to enhance the survival, colonization potential, and functional activity of probiotic strains during storage and gastrointestinal transit. These substrates may also stimulate the growth of resident beneficial taxa, including *Bifidobacterium adolescentis*, *Lactobacillus acidophilus*, and *Faecalibacterium prausnitzii*, thereby promoting the production of SCFAs and other bioactive metabolites associated with intestinal health [24]–[26].

Compared with traditional yogurt, synbiotic yogurt are designed to create synergistic interactions between administered microorganisms and fermentable substrates. This combination may improve probiotic persistence, enhance microbial diversity, and strengthen microbiota-mediated metabolic functions, although the magnitude and durability of these effects remain dependent on strain selection, substrate composition, and host factors. Emerging studies employing synbiotic yogurt formulations have reported enhanced survival of probiotic strains during storage and gastrointestinal transit, increased SCFAs production, and modulation of specific gut taxa in animal models and preliminary human studies [27].

### 3.4. Fortified yogurt

Fortified yogurt refers to yogurt products enriched with additional bioactive compounds that are not primarily intended to function as microbial substrates, such as polyphenols, plant extracts, vitamins, or minerals, without necessarily altering the core microbial component. These fortificants may act through antioxidant, anti-inflammatory, and metabolic pathways in synergy with microbial fermentation products.

Among these fortificants, plant polyphenols have received considerable attention. Polyphenol-rich extracts derived from fruits such as bilberry and blackcurrant are increasingly incorporated into yogurt to enhance antioxidant capacity and functional value. Beyond their direct antioxidant properties, these compounds may influence gut microbial ecology by promoting the growth of beneficial taxa such as *Akkermansia muciniphila* and *Bifidobacterium longum* while suppressing potentially detrimental microorganisms, including *Enterococcus faecalis* [28]–[31]. Polyphenols may also modulate host inflammatory pathways through mechanisms involving attenuation of NF-κB signaling and reduction of oxidative stress [32].

Fortified yogurts may additionally contain vitamins, minerals, and omega-3 fatty acids to address specific nutritional deficiencies or support targeted health outcomes. These ingredients primarily exert host-directed physiological effects but may also indirectly influence gut microbial metabolism through alterations in nutrient availability.

Another emerging category of fortified yogurt incorporates postbiotics, including inactivated microbial cells, or extracellular polysaccharides. Examples include preparations derived from *Pediococcus lactis* and *Lactobacillus paracasei*. Unlike live probiotics, postbiotics do not require microbial viability and may offer advantages in terms of product stability and safety while retaining immunomodulatory and metabolic activities [33]. However, evidence supporting the efficacy of specific postbiotic-fortified yogurts remains relatively limited.

### 3.5. Non-dairy yogurt analogs

Non-dairy yogurts, such as those based on soy milk, represent a distinct category in which the substrate and resulting microbial ecosystem differ from dairy yogurts [34]. Plant-based yogurts often lack lactose and have alternative protein and carbohydrate matrices, requiring adaptation of starter cultures for fermentation and texture development. While this plant-based yogurts may offer benefits for individuals with lactose intolerance or dairy allergies, their microbial dynamics, nutrient profiles, and host interactions are not directly comparable to dairy yogurt, and evidence regarding their effects on gut microbiota remains more limited.

Similarly, certain regional fermented milk products, such as Tibetan yak yogurt, are produced from non-bovine milk sources (e.g., yak milk) using traditional, often undefined, microbiota. These products harbor unique microbial lineages adapted to high-altitude conditions, including specific *Lactobacillus* and *Bifidobacterium* strains. While they share some functional similarities with traditional yogurt, their microbial composition, metabolic profiles, and host interactions are not directly comparable. Evidence regarding their effects on gut microbiota remains more limited.

## 4. Mechanistic pathways linking yogurt consumption to host health

**Figure 2.**
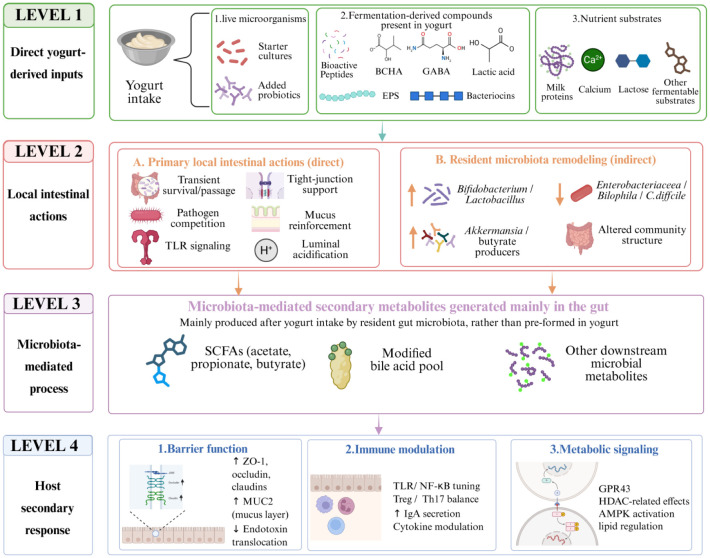
Mechanistic hierarchy of yogurt effects on host health. Yogurt intake delivers live microorganisms, fermentation-derived compounds, and nutrient substrates that act directly on the gut. These inputs modulate intestinal barrier integrity, mucus, and local immune signaling while reshaping resident microbiota. Microbiota-mediated secondary metabolites, produced mainly after consumption, further influence host metabolism, immune responses, and signaling pathways. The framework distinguishes direct yogurt-borne effects, microbiota-mediated actions, and host secondary responses.

The biological effects of yogurt should not be interpreted as a single linear pathway. Rather, yogurt consumption initiates a layered sequence of interactions involving yogurt-borne microorganisms and fermentation-derived compounds, the resident intestinal microbiota, microbiota-derived secondary metabolites, and host-mediated downstream responses. To improve mechanistic clarity, in this section, we distinguish four interconnected levels: (1) Direct yogurt-derived inputs, including live starter cultures, added probiotic strains, fermentation-derived compounds, and the dairy matrix; (2) primary local intestinal actions and resident microbiota remodeling; (3) microbiota-mediated secondary metabolites generated mainly after yogurt intake; and (4) host secondary responses involving barrier function, immune regulation, and metabolic signaling. Figure 2 summarizes this hierarchical framework and is supported by the mechanistic evidence discussed in the following sections and highlights the impact of yogurt consumption on gut health.

### 4.1. Direct yogurt-derived inputs and primary local intestinal actions

Traditional and probiotic yogurts deliver live microorganisms, fermentation-derived compounds, and dairy matrix components directly to the gastrointestinal tract. Although most yogurt-associated bacteria do not permanently colonize the gut, they may survive transiently and interact with epithelial cells, mucus, resident microbes, and luminal substrates during passage [35],[36]. The yogurt matrix, including milk proteins, fat globule components, calcium, and semi-solid gel structure, can protect bacteria from gastric acid and bile stress and may improve delivery of viable microorganisms to the intestine [37].

These direct inputs can support local intestinal function. In murine models, *L. reuteri* fermented yogurt increased jejunal claudin-1 and occludin expressions after challenged with enterotoxigenic *Escherichia coli*, reducing intestinal permeability and plasma diamine oxidase levels [38]. Mechanistically, probiotic and yogurt-associated bacteria may act through TLR2-dependent signaling, which promotes ZO-1 phosphorylation and occludin redistribution to tight-junction complexes [39],[40]. Certain *S. thermophilus* strains also show enhanced adhesion to intestinal epithelial cells and competitive exclusion of pathogens such as *Listeria monocytogenes* [41]. In allergic murine models, yogurt-associated *Lactobacillus* strains increased mucin synthesis and reduced *Staphylococcus aureus* adhesion [42]. These findings support direct epithelial and mucosal effects, although most evidence remains preclinical.

Yogurt-derived local actions also include luminal acidification and pathogen competition. Lactic acid is generated during yogurt fermentation and is present in the product, unlike SCFAs, which are mainly produced later in the gut. Together with bacteriocins, microbial surface molecules, and milk-fat globule membrane components, yogurt-derived bacteria may reduce pathogen adhesion and suppress acid-sensitive *Enterobacteriaceae* [43],[44]. These effects should be interpreted mainly as short-term local interactions rather than definitive evidence of durable microbiota restructuring.

### 4.2. Resident microbiota remodeling as an indirect pathway

In parallel with direct local actions, yogurt intake may reshape the resident gut microbiota. This is an indirect process because the outcome depends on baseline microbiota composition, diet, host factors, and yogurt formulation. Human intervention studies have reported increased fecal *Bifidobacterium* and *Lactobacillus* after daily yogurt intake [45]. Detection of *L. delbrueckii* subsp. *bulgaricus* in feces of yogurt consumers also suggests survival through gastrointestinal transit, but this should be interpreted as transient passage rather than stable colonization [46].

Probiotic yogurts may strengthen microbiota-mediated effects by adding strains with acid tolerance, bile resistance, or adhesion capacity. For example, *Lactobacillus paracasei* ZFM54 co-fermented yogurt altered yogurt metabolomic profiles and was associated with enrichment of butyrogenic *Ruminococcus* and immunomodulatory *Alistipes*, while suppressing opportunistic *Enterobacteriaceae* [45]. *Bifidobacterium animalis* subsp. *lactis* BB-12 supplementation has also been shown to enhance fecal *Bifidobacterium* loads in children [47].

Synbiotic yogurts add another layer because prebiotic substrates such as inulin, FOS, GOS, or konjac-derived oligosaccharides can feed resident beneficial taxa and support microbial cross-feeding. These substrates may promote *Bifidobacterium longum*, *Lactobacillus acidophilus*, and *Faecalibacterium prausnitzii*, thereby increasing downstream microbial metabolites such as SCFAs [48]. Therefore, synbiotic yogurt should not be treated as equivalent to traditional yogurt; its effects are partly substrate-driven and microbiota-mediated.

Evidence from dysbiosis models further supports yogurt-related microbiota remodeling but remains largely preclinical. *L. plantarum* Lp3 isolated from Tibetan yak yogurt was associated with reduced Proteobacteria expansion and increased *Bifidobacterium adolescentis* [49]. BB-12-fortified yogurt was associated with partial preservation of microbial diversity during antibiotic exposure and restricted *C. difficile* expansion [50]. Plateau-yogurt-derived *Pediococcus acidilactici* BT36 increased *Lachnospiraceae* abundance and increased cecal butyrate in chromium-exposed mice [51]. These data suggest potential ecological effects.

### 4.3. Yogurt-derived metabolites versus microbiota-derived secondary metabolites

Yogurt-derived metabolites are produced during fermentation and may be present in the consumed product. These include lactic acid, BCHAs, bioactive peptides, EPS, GABA in specific fermented products, vitamins, and bacteriocins. Microbiota-derived secondary metabolites, by contrast, are produced mainly by resident gut microbes after yogurt consumption. These include SCFAs and modified bile acid pools.

BCHAs are representative yogurt-derived postbiotics. They are generated during fermentation through branched-chain amino acid metabolism by *L. delbrueckii* subsp. *bulgaricus* and selected *Lactobacillus casei* strains. In diet-induced obese mice, yogurt-associated BCHAs activated hepatic AMPK signaling, increased fatty acid β-oxidation, and reduced hepatic triglyceride accumulation. BCHAs also showed antioxidant effects, reducing malondialdehyde and protecting intestinal villi from oxidative injury in preclinical models [51],[52].

Bioactive peptides are released from casein and whey proteins during fermentation, especially in traditional, high-protein, or strain-specific yogurts [53]. These peptides may exert ACE-inhibitory, antimicrobial, antioxidant, and immunomodulatory effects, although their activity depends on peptide sequence and digestive stability [54]. EPS, produced by certain lactic acid bacteria, contribute to yogurt viscosity and may enhance bacterial survival, adhesion, and immune interaction [55],[56]. EPS may also act as fermentable substrates for resident microbes, thereby linking direct yogurt-derived compounds to downstream microbiota-mediated effects [57].

GABA should be described as product-specific rather than universal. In soy yogurt fermented with GABA-producing *Lactiplantibacillus plantarum* GA30, GABA production was associated with improved pancreatic β-cell recovery and early-phase insulin secretion in diabetic mice [34]. Similarly, vitamins such as riboflavin, folate, and vitamin B_12_, as well as bacteriocins, such as nisin, may be produced during fermentation, but their concentrations and biological relevance vary by strain and product type [58]–[60].

SCFAs should be placed in the microbiota-derived category. Acetate, propionate, and butyrate are mainly produced in the intestine through fermentation of residual lactose, GOS, protein-derived substrates, and other fermentable compounds by resident microbiota [61],[62]. Yogurt may increase SCFAs indirectly by delivering fermentable substrates, enriching beneficial taxa, or supporting cross-feeding networks. For example, BB-12-supplemented yogurt increased propionate production and was accompanied by improved insulin sensitivity in obese individuals [63],[64]. Thus, SCFAs are best interpreted as microbiota-mediated secondary metabolites rather than pre-formed yogurt metabolites.

Bile acid modulation also represents a mixed pathway. Yogurt-derived bacteria may contribute bile-salt hydrolase activity, but the final bile acid pool is largely shaped by resident microbial metabolism [65],[66]. Therefore, changes in secondary bile acids, such as reduced fecal 7-ketolithocholic acid, should be described as microbiota-mediated bile acid remodeling supported by yogurt-associated microbial activity rather than as a direct yogurt-derived metabolite effect.

Although many of these compounds are produced by resident gut microbiota, yogurt intake can increase their levels by enriching beneficial bacteria and providing fermentable substrates.

### 4.4. Host-mediated downstream responses

Direct yogurt-derived inputs and microbiota-mediated metabolites converge on host barrier, immune, and metabolic pathways. Barrier reinforcement involves direct microbial contact and microbiota-derived metabolites. Yogurt-associated bacteria may increase tight-junction proteins and mucus production, while butyrate generated by resident microbiota supports epithelial energy metabolism and HDAC-related regulation of barrier genes [37]–[42]. The downstream outcome includes increased ZO-1, occludin, claudins, and MUC2, with reduced permeability and endotoxin translocation.

Immune modulation is strain- and context-dependent. Yogurt-derived microbial ligands can regulate TLR/NF-κB signaling. In immunosuppressed models, yogurt fermented with *Bifidobacterium* and containing D-lactate activated TLR4/MyD88/NF-κB signaling and promoted cytokine production [67],[68]. Conversely, *S. thermophilus* 19 attenuated inflammation in septic models by inhibiting NF-κB and reducing TNF-α, IL-1, and IL-6 [69]. Soymilk yogurt fermented with *Pediococcus pentosaceus* TOKAI 759m also suppressed inflammatory cytokine production in macrophage-related models [70]. These examples indicate that yogurt may enhance immune defense under deficient conditions while limiting excessive inflammation in inflammatory settings [71].

Yogurt-associated lactic acid bacteria also influence adaptive and mucosal immunity. *L. bulgaricus* TCI904 improved immune balance in obese models [72], while yogurt containing *L. bulgaricus* 151 and *S. thermophilus* MK-10 increased regulatory T cells and balanced Th1/Th2 responses in DSS-induced colitis [73]. Fermented milk containing *L. casei* DN-114001 increased IgA-producing plasma cells and secretory IgA levels [74], and clinical studies suggest that live starter cultures may be important for immune-marker changes [75].

Metabolic signaling reflects yogurt-derived and microbiota-derived pathways. BCHAs can activate AMPK-mediated fatty acid oxidation [76], EPS may support butyrate-producing bacteria and AMPK/ACC signaling [77], and microbiota-derived SCFAs act through GPR43, HDAC inhibition, and gut-brain signaling to regulate glucose metabolism, inflammation, and epithelial function [78],[79]. Product-specific bioactives, such as GABA in soy yogurt, may also influence insulin secretion and pancreatic β-cell function [80].

Overall, yogurt acts through an integrated hierarchy. First, yogurt provides live microorganisms, fermentation-derived compounds, and matrix substrates. Second, these inputs exert local intestinal effects and interact with resident microbiota. Third, resident microbes generate secondary metabolites such as SCFAs and modified bile acids after yogurt consumption. Finally, direct and indirect signals converge on host barrier integrity, immune regulation, and metabolic signaling. This framework explains why different yogurt categories, such as traditional, probiotic, synbiotic, fortified, soy-based, or regional fermented yogurts, may produce different biological effects. As summarized in Table 1, the current literature is organized according to major mechanistic domains of yogurt action, and for each domain the table further categorizes the type of supporting evidence and the corresponding level of evidence strength, allowing a structured evaluation of both mechanistic pathways and their possibility of translation.

**Table 1.** Mechanisms, evidence type, and evidence level.

Mechanistic domain	Main pathway	Yogurt category mainly involved	Predominant evidence type	Evidence strength	References
Direct yogurt-derived microbial input	Delivery of starter cultures; transient gastrointestinal survival; interaction with epithelial cells, mucus, and resident microbes	Traditional yogurt; probiotic yogurt	*In vitro* studies, animal models, short-term human studies	Low–Moderate	[13],[14],[16],[17]
Primary epithelial barrier support	Increased tight-junction proteins, mucus reinforcement, reduced epithelial permeability	Traditional fermented yogurt; probiotic yogurt	Mainly animal models and *in vitro* studies	Low–Moderate	[38]–[42]
Pathogen competition and luminal acidification	Lactic acid, bacteriocins, microbial adhesion, and receptor competition suppress pathogen adhesion or growth	Traditional yogurt; probiotic yogurt	*In vitro* studies, animal models, short-term intervention data	Low–Moderate	[41],[44]
Resident microbiota remodeling	Increased *Bifidobacterium, Lactobacillus*, *Akkermansia*, and butyrate-producing taxa; decreased pathobionts	Probiotic yogurt; synbiotic yogurt; regional fermented yogurt	Animal models, short-term human interventions, observational studies	Moderate	[45]–[51]
Microbiota-derived secondary metabolites	SCFAs generated mainly after ingestion from residual lactose, GOS, protein-derived substrates, and other fermentable components	Synbiotic yogurt; probiotic yogurt; traditional yogurt	Animal models, observational studies, short-term human interventions	Moderate	[61],[62],[64],[67]
Immune modulation	TLR/NF-κB tuning, cytokine modulation, T-cell balance, and IgA-mediated mucosal immunity	Traditional yogurt; probiotic yogurt; soy yogurt	*In vitro* studies, animal models, limited clinical studies	Low–Moderate	[68]–[75]

Evidence strength definitions: Low = mainly *in vitro* evidence, animal models, or single small studies; Low–Moderate = consistent preclinical evidence with limited human support; Moderate = supported by multiple preclinical studies and at least some human observational or intervention evidence. High-level evidence was not assigned because long-term, adequately powered randomized controlled trials remain limited for most outcomes.

## 5. Health effects in specific conditions

Accumulating evidence indicates that yogurt-induced modulation of gut microbiota produces downstream benefits that extend beyond the intestinal lumen [81]–[86], influencing multiple organs through the gut–systemic axis. However, the strength of evidence varies substantially across disease areas and yogurt categories. Researchers mostly focus on three domains: neurological, gastrointestinal, and hepatic health. In the following sub-sections, we summarize these findings and highlight the microbial or bioactive mediators involved in each context. Importantly, many of the studies cited in this section are observational or involve multi-component dietary patterns, making it difficult to attribute observed effects solely to yogurt. Confounding variables such as overall dietary quality, lifestyle factors, socioeconomic status, and co-interventions may contribute to or modify the reported associations. Therefore, while the evidence suggests potential benefits, causality and product-specific attribution should be interpreted with caution. To facilitate interpretation of this heterogeneous evidence base, Table 2 provides a structured overview of reported health effects across different disease conditions, categorizing them according to affected organ systems, predominant microbiota- or metabolite-mediated mechanisms, and the corresponding level of evidence.

### 5.1. Neurological and psychological disorders (gut-brain axis modulation)

Emerging evidence suggests that yogurt and yogurt-like fermented products may be associated with neurological and psychological outcomes through gut–brain-axis-related pathways [87]. By inducing beneficial microbiota shifts, yogurt may alleviate cognitive decline, depression, and exercise-related psychological fatigue [88].

**Animal evidence.** High altitude Tibetan fermented yogurt (rich in diverse probiotics) significantly improved spatial learning and object recognition in Alzheimer's disease (AD) transgenic mice (APP/PS1 model). After 20 weeks of intervention, Aβ plaque deposition in the hippocampus and cortex decreased, correlating with elevated *Bacteroides* and *Faecalibacterium* abundance. Notably, *Mucispirillum* reduction and *Muribaculum* enrichment predicted cognitive improvement, confirming microbiota mediated neuroprotection [89]. Similarly, soy yogurt fermented with *Pediococcus pentosaceus* TOKAI 759m mitigated high-fat diet-induced neuroinflammation in mice, reducing hippocampal IL-6 and TNF-α, while proteomics revealed up-regulation of synaptophysin and mitochondrial ATP-synthase sub-units, supporting improved novel-object recognition scores [90].

**Human evidence.** Population-level studies reinforce these findings. NHANES 2005–2016 showed that probiotic-yogurt consumers were associated with lower odds of depression, with improvements in anhedonia, fatigue, and poor sleep. In depressed individuals, this was associated with lower all-cause mortality, particularly among males aged 40–60 years [91]. This study supports an association between fermented or microbiota-targeted foods and mental health outcomes. In a double-blind trial with female athletes, 8-week BB-12 yogurt decreased Athlete Burnout Questionnaire scores, paralleled by a enrichment of *Bifidobacterium*, suppression of *E. coli* and a rise in faecal L-arginine that negatively correlated with burnout dimensions [92]. This provides preliminary human intervention evidence for a strain-specific probiotic yogurt, but the population and endpoint were narrow, and broader clinical translation requires larger trials.

### 5.2. Colorectal cancer (CRC)

Yogurt intake has been associated with lower colorectal cancer or colorectal neoplasia risk in several observational studies, while mechanistic evidence comes largely from animal and molecular studies.

**Animal and mechanistic evidence.** A synbiotic combination containing *Lactobacillus gasseri* 505 and *Cudrania tricuspidata* leaf extract showed protective effects in colitis-associated colorectal cancer models [93]. However, this intervention is a synbiotic formulation rather than traditional yogurt. Additional preclinical evidence suggests that yogurt-derived or probiotic-associated mechanisms may include pathobiont exclusion, inhibition of *Fusobacterium nucleatum*, suppression of IL-6/IL-8 signaling, barrier enhancement, D-lactate-related inhibition of PI3K–AKT–β-catenin signaling, and nisin-mediated down-regulation of cyclin D1 [94]–[97].

**Human evidence.** Prospective cohort studies reveal a dose-dependent protective association [98]. In the Nurses' Health Study and Health Professionals Follow-Up Study, regular yogurt intake was associated with reduced proximal colon cancer risk, with maximal benefit observed after 16–20 years of consistent intake [99]. A 2025 molecular pathological epidemiology study further stratified this effect by tumor microbiome: Yogurt intake (≥2 servings/week) was associated with lower CRC incidence only in *Bifidobacterium* positive tumors but not in *Bifidobacterium* negative tumors [100]. In Chinese populations, diets high in yogurt, vegetables, and fruits were associated with lower colorectal neoplasm risk in individuals with *Bacteroides*-dominant enterotypes [101]. Because yogurt was part of a broader dietary pattern, attribution to yogurt alone is limited.

### 5.3. Inflammatory bowel disease (IBD)

Yogurt may influence IBD-related pathways, and exert therapeutic effects in IBD through microbiota modulation, immunoregulation, and barrier reinforcement [102].

**Animal evidence using yak-yogurt-derived strains.** In ulcerative colitis (UC), *Lactiplantibacillus plantarum* DACNJS22, isolated from traditional yak yogurt, significantly alleviated DSS-induced colitis in mice by suppressing pro-inflammatory cytokines, upregulating tight junction proteins, and enriching SCFA-producing *Faecalibaculum* while reducing pathogenic *Enterobacteriaceae* [103]. This strain exhibited strong acid/bile tolerance and intestinal adhesion. Similarly, *Lacticaseibacillus rhamnosus* G7 from herdsmen yogurt reduced disease activity index (DAI) scores and mitigated colon shortening while increasing anti-inflammatory IL-10 and suppressing *Bacteroides* [104].

**Human intervention evidence.** A double blind trial in IBD patients consuming probiotic yogurt for 8 weeks increased fecal *Lactobacillus*, *Bifidobacterium*, and *Bacteroides*, correlating with improved intestinal function [105]. Additional reports suggest that *Bifidobacterium* fortified formulations, particularly when combined with glutamine or other supportive ingredients, may contribute to symptom improvement or remission in some IBD patients [105],[106].

***In vitro* immune-cell evidence.** Peripheral blood mononuclear cells from UC patients stimulated with *Bifidobacterium animalis* BB-12 and *Lactobacillus acidophilus* LA-5 showed time-dependent cytokine modulation, with IL-10 and TGF-β peaking earlier and TNF-α and IFN-γ decreasing later [107]. This supports immunomodulatory potential of probiotic yogurt strains but does not by itself demonstrate clinical efficacy.

### 5.4. Functional gastrointestinal disorders (FGIDs)

Functional gastrointestinal disorders, particularly constipation and IBS-related symptoms, have been studied using synbiotic, probiotic, and multi-strain yogurt formulations. Compared with other disease domains, this area includes more human intervention evidence, but studies remain heterogeneous in formulation, duration, and endpoints.

**Animal evidence.** Probiotic and synbiotic yogurt formulations have been investigated for functional gastrointestinal symptoms, particularly constipation, through proposed effects on motility, microbial fermentation, and gut–brain signaling. In constipated mice, synbiotic yogurt combining konjac-mannan-oligosaccharides with *Bifidobacterium animalis* BB-12 elevates pro-kinetic motilin while suppressing inhibitory nitric-oxide, activating SCF/c-Kit signaling in interstitial cells of Cajal, and upregulating serotonin-4 receptors [108].

**Human intervention evidence.** *B. animalis* subsp. *lactis* MN Gup yogurt elevated fecal acetate in constipated adults, improving stool frequency and consistency. Metagenomic analysis linked this to enriched acetate producers (*Ruminococcaceae*_UCG 002/005) [109]. A three-strain yogurt (*L. bulgaricus*, *S. thermophilus*, *B. lactis*) further raises Shannon diversity while moderating excessive SCFAs in slow-transit constipation patients, indicative of restored fermentation homeostasis [110]. Randomized trials have reported improvements in selected symptom scores. Four-week ingestion of a seven-species, six-fiber yogurt in 86 constipated adults reduced PAC-SYM straining scores and incomplete-evacuation, improved quality-of-life (PAC-QOL) and enriched stool bioactive peptides that positively correlated with bowel-movement frequency [111].

**Mechanistic evidence.** In terms of mechanism, propionate and butyrate generated by *L. casei*-fermented milk activate GPR41/43 on enteric neurons, suppress endothelin-1, and enhance peristalsis [112], while folate-producing *Lactobacillus* in synbiotic yogurt correct one-carbon metabolism in IBS-D, cutting serum homocysteine [113]. Thus, yogurt may function as a “neuro-fermentative” intervention restoring motility, reducing inflammation, and re-establishing microbial balance [114].

### 5.5. Liver diseases

Across non-alcoholic and alcoholic liver disease models, yogurt or yogurt-related interventions have been associated with improvements in selected hepatic and inflammatory markers [115].

**Animal evidence.** These changes were accompanied by *Bifidobacterium* driven suppression of endotoxemia and enhanced fatty acid oxidation, with greater changes than the milk comparator in some metabolic outcomes. In line with these findings, probiotic yogurt supplementation has been shown in a high-fat diet golden hamster model to regulate gut microbiota homeostasis and alleviate hepatic steatosis and liver injury [116]. High-protein yogurt further reduced hepatic triglycerides in high-fat diet mice via *Lactobacillus* bloom, LPS reduction, and restored GLP-1 amide [117]. Combining yogurt with intermittent caloric restriction increased *Lactobacillus*, upregulated hepatic CPT1A, and downregulated SREBP-1c [118], while probiotic yogurt (*Lacticaseibacillus rhamnosus* HF01) enriched *Muribaculaceae*, raised cecal butyrate, and activated AMPK/PGC-1α to accelerate mitochondrial fat oxidation [119]. For alcohol induced liver injury (ALD), *Lactobacillus*-derived extracellular vesicles (LAB EVs) reduced serum ALT/AST in mice by activating the Nrf 2 antioxidant pathway and restoring gut microbiota diversity. LAB EVs also suppressed TLR4/NF-κB driven inflammation [120].

**Human RCT evidence.** In nonalcoholic fatty liver disease (NAFLD), a 24 week RCT in obese women revealed that daily consumption of 220 g conventional yogurt significantly reduced hepatic fat fraction and intrahepatic lipids while decreasing serum LPS and pro-inflammatory cytokines [121].

**Table 2.** Summary of yogurt-associated mechanisms and evidence levels.

Diseases	Mechanism	Effect	Predominant evidence type	Evidence strength	References
Neurological and psychological disorders	• Gut-brain axis modulation • Anti-neuroinflammation	• Decrease Aβ plaque (AD mice) • Lower odds of depression • improved cognitive/psychological outcomes	Animal models + small RCTs	Low–Moderate	[88]–[92]
Colorectal cancer	• Enterotype specificity • Pathobiont exclusion (↓*Fusobacterium*) • Tumour-suppressive metabolites (D-lactate, nisin, SCFA)	• Reduced tumor multiplicity in mice • association with lower CRC risk in cohort studies	Animal models + observational	Moderate	[93]–[101]
Inflammatory bowel disease	• Cytokine rebalancing • Barrier repair (occludin/claudin-1↑) and SCFA restoration • Pathogen exclusion (↓*Enterobacteriaceae*)	• symptom improvement or remission-related outcomes in mild-moderate UC • Patient-reported symptom relief	*in vitro* + small RCTs	Low–Moderate	[102]–[107]
Functional gastrointestinal disorders	• Pro-kinetic neuropeptides and 5-HT4 up-regulation • Microbiota diversification and excess SCFA moderating	• ↓straining, ↓incomplete evacuation • Improve defecation frequency • Reduce homocysteine (IBS-D)	Animal models + pilot human interventions	Low–Moderate	[108]–[113]
Liver diseases	• ↓LPS, Desulfovibrio and ↑butyrate • Nrf-2 induction and bile-acid deconjugation	• ↓hepatic fat fraction • ↓hepatic TG in mice • ↓ALT/AST in mice	Animal models + small clinical trials	Low–Moderate	[115]–[121]

Evidence strength definitions: Low = mainly *in vitro* evidence, animal models, or single small studies; Low–Moderate = consistent preclinical evidence with limited human support; Moderate = supported by multiple preclinical studies and at least some human observational or intervention evidence. High-level evidence was not assigned because long-term, adequately powered randomized controlled trials remain limited for most outcomes.

**Figure 3.**
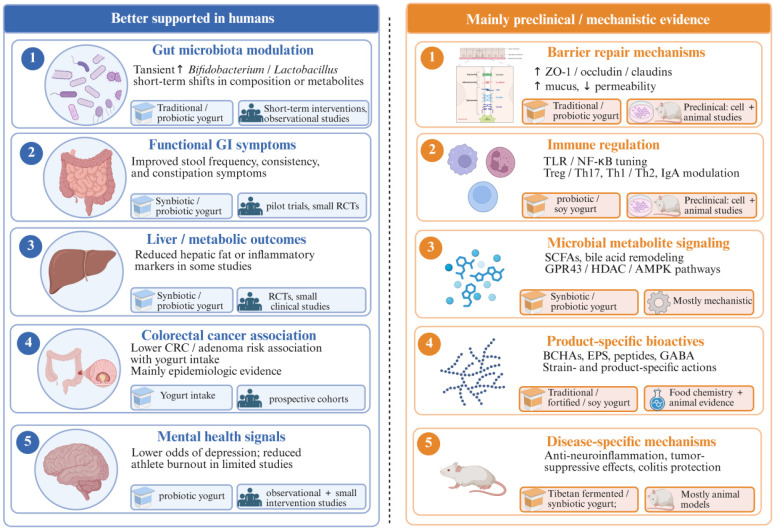
Translational evidence map of yogurt-associated health effects. “Human evidence” includes RCTs, observational studies, and human intervention trials, while “preclinical evidence” includes animal models and *in vitro* studies.

To further clarify the translational relevance of the evidence summarized above, we distinguish findings that have been reported in human studies from mechanisms that remain mainly supported by preclinical or mechanistic data. As shown in Figure 3, human evidence is comparatively stronger for short-term gut microbiota modulation, functional gastrointestinal symptom improvement, selected liver/metabolic outcomes, colorectal cancer risk associations, and preliminary mental health-related outcomes. By contrast, many proposed mechanisms, including epithelial barrier repair, immune-cell reprogramming, microbial metabolite signaling, and disease-specific pathways such as anti-neuroinflammation or colitis protection, remain largely dependent on animal models, *in vitro* systems, or product-specific mechanistic studies. This evidence map emphasizes that yogurt should not be interpreted as a homogeneous intervention and that translational conclusions must consider yogurt category, study design, and evidence level.

## 6. Clinical translation: Challenges and personalized approaches

### 6.1. Inter-individual variability and precision nutrition

Individual responses to yogurt intake appear to be influenced by host-specific factors, including baseline microbiome composition, genetic polymorphisms, and dietary patterns. Large scale cohort studies reveal that individuals with initially low *Bifidobacterium* abundance exhibit a greater increase in microbial diversity after yogurt intervention compared to those with higher baseline levels. Gender-related differences are also evident: Females show stronger associations between yogurt intake and *Lactobacillus casei* subgroup enrichment, while males demonstrate greater reductions in *Enterobacteriaceae* [122]. Genetic variants further modulate responses, as non-secretors derive less *Akkermansia* mediated barrier enhancement from dairy consumption [123]. These findings underscore the need for precision nutrition strategies integrating metagenomic profiling and host genomics to optimize yogurt interventions.

### 6.2. Standardization of yogurt products

Commercial yogurt products are produced under established food safety and quality standards, which typically regulate starter cultures, fermentation conditions, and minimum viable bacterial counts. These standards ensure product safety and consistency at the manufacturing level. However, substantial variability exists in microbial composition, metabolite content, and functional activity across brands and production systems. Factors such as milk source, starter strain selection, fermentation temperature and duration, and post-fermentation processing can influence the abundance and viability of probiotic strains, as well as the levels of key metabolites, including SCFAs, BCHAs, and EPS. Moreover, many commercial products rely on a limited number of well-established starter strains to ensure reproducibility and regulatory compliance. While this approach supports industrial stability, it may also constrain microbial diversity and functional specificity. Regional production practices further contribute to heterogeneity: High-altitude Tibetan yogurts naturally harbor unique *Lactobacillus* and *Bifidobacterium* lineages adapted to hypoxic conditions, while Nordic yogurts may be enriched in propionate-producing *Propionibacterium*. These geographical and processing differences create distinct “functional fingerprints”, complicating cross-study comparisons and potentially leading to over- or underestimation of health benefits. Therefore, improved standardization may help transform yogurt research from heterogeneous product-based observations into more reproducible evidence on functional food interventions. Current retail yogurts are insufficiently standardized for direct comparison across mechanistic or clinical studies. Flow-cytometric and culture analyses reveal that labeled counts frequently overestimate true viability due to strain dependent viability loss [124]. Concurrently, metabolite concentrations can vary considerably between brands, reflecting differences in fermentation control, pH drift, and strain-specific biosynthetic capacity. Synbiotic formulations, which combine probiotics with prebiotics, can improve microbial engraftment but require stability validation under storage conditions.

To explore the potential for yogurt to become a more reproducible functional food, we propose the following directions for future efforts: (i) Future studies could explore bioactivity-informed labels that report, in addition to viable cells, a minimum panel of post-biotics quantified by targeted LC-MS/MS; (ii) real-time stability protocols including 6-week shelf-life (25 °C) and 28-day cold-chain simulations with quarterly flow-cytometric enumeration and metabolite profiling; and (iii) strain-level documentation to improve transparency and comparability across studies. Adoption of these considerations may facilitate yogurt from a variable commodity into a reproducible, evidence-based functional food suitable for prescriptive use in microbiome-targeted therapy.

### 6.3. Considerations for infants and elderly

Yogurt interventions should be adapted according to developmental stage, baseline microbiota, and socio-economic context to maximize functional efficacy. Moreover, several safety and tolerance issues specific to these groups require explicit consideration to avoid oversimplified benefit narratives.

#### 6.3.1. Infants and toddlers

Moderate daily intake of yogurt has been associated with reduced incidence of infectious diarrhea and lowers the risk of atopic dermatitis, likely mediated by *Bifidobacterium*-driven IgA enhancement [125]. Observational evidence also suggests that moderate consumption (3–4 servings/week) at age 1 year may be associated with lower risk of developmental delay at age 3 years, while excessive intake (≥5 servings/week) diminishes benefits [126], probably due to micronutrient displacement and excess caloric intake. Moreover, yogurt is generally well tolerated, with randomized trials reporting adverse-event rates comparable to non-probiotic controls [127].

However, infants and toddlers are not a homogeneous group, and distinct physiological characteristics may increase risk. The small intestine's lactase activity in young infants may be insufficient to digest residual lactose in dairy products, potentially causing gas, bloating, or diarrhea in sensitive individuals. Primary lactose intolerance is uncommon before 2–3 years of age, but symptoms can occur due to immature gut enzyme activity or secondary lactase deficiency due to enteric illness [128]. Also, immune-mediated allergy to milk proteins is relatively common in children and can lead to digestive symptoms, or in rare cases, life-threatening anaphylaxis. Avoidance of milk and milk-containing products such as yogurt is recommended in affected infants. Furthermore, many commercial flavored yogurts contain significant added sugars, potentially contributing to excessive sugar burden.

Overall, while certain well-tolerated, low-sugar yogurt introduced after appropriate developmental milestones may be included as part of a diverse complementary feeding strategy, careful monitoring for intolerance, allergic reactions, and nutrient balance is warranted, and recommendations should be individualized.

#### 6.3.2. Elderly (>65 years)

Yogurt consumption in older adults has been associated with improved dietary quality and may be a convenient source of protein and micronutrients. Some observational data suggest associations with higher intake of calcium, riboflavin, and decreased prevalence of nutrient deficiencies [129]. Hospitalized older adults consuming probiotic yogurt containing *B. lactis* LKM512 maintain elevated fecal *Bifidobacterium* levels for at least two weeks post-intervention, correlating with improved defecation frequency [130]. Strains such as *B. lactis* GCL2505 show longer ecological persistence than heat-inactivated equivalents, making live formulations preferable when not contraindicated [131].

However, as mentioned above, lactose intolerance and sugar burden also need attention in the elderly population, particularly in older adults with type 2 diabetes or metabolic syndrome. Moreover, yogurt's acidic pH and active cultures may interact with gastrointestinal physiology in individuals taking multiple medications or with underlying gastrointestinal sensitivity. Importantly, older adults with immunosenescence or those in hospital settings may have increased susceptibility to opportunistic infections. Although rare, cases of probiotic-related bacteremia or fungemia have been reported with live cultures in immunocompromised hosts [132]; therefore, strain-specific safety data from healthy populations are necessary.

For these reasons, yogurt intake in the elderly should consider individual tolerability, metabolic status, and product formulation. Blanket recommendations without context may overlook important safety issues.

## 7. Limitations and controversies

Despite the growing evidence supporting the beneficial effects of yogurt on gut microbiota and host health, several limitations and ongoing controversies should be acknowledged. First, substantial heterogeneity exists across studies in terms of yogurt formulation, microbial composition, fermentation process, dosage, and duration of intake. Many studies fail to clearly define strain identity, microbial viability, or metabolite profiles, making it difficult to compare results or attribute observed effects to specific components. As a result, inconsistencies in reported microbiota and health outcomes may reflect methodological variability rather than true biological differences. Second, the interpretation of microbiota changes remains challenging. Increases in microbial diversity or the abundance of specific microbiota are often assumed to be beneficial; however, such changes do not necessarily translate into functional or clinical improvements. Moreover, the transient detection of yogurt-associated microorganisms in fecal samples raises debate as to whether observed effects reflect true ecological integration or short-term exposure. Functional outcome, including microbial metabolites and host responses, are therefore essential but remain underrepresented in many studies. Third, host-related factors introduce additional complexity and controversy. Baseline microbiota composition, habitual diet, metabolic status, and immune function can strongly influence individual responses to yogurt consumption, contributing to variable or null findings in population-level analyses. This inter-individual variability challenges the generalizability of current conclusions and highlights the limitations of this dietary recommendation. Finally, much of the evidence is derived from short-term interventions or observational studies, which limits causal inference. While randomized controlled trials provide stronger evidence, many are of insufficient duration to capture long-term microbial and physiological changes. Together, these limitations underscore the need for more standardized, function oriented, and longitudinal research approaches, as discussed in the following section on future research directions.

## 8. Future research directions

Future research should further clarify the strain-specific mechanisms through which yogurt influences the gut microbiota, as different microbial strains and fermentation processes may produce distinct biological effects. To address this, long-term RCTs using yogurt products with clearly defined and standardized microbial compositions are needed. Extended intervention and follow-up periods will be important to distinguish short-term microbiota changes from lasting changes in gut microbial structure. Furthermore, improved standardization of yogurt and reporting protocols is essential to enhance reproducibility across studies. This effort can be supported by integrated multi-omics approaches, such as metagenomics and metabolomics, combined with host-related measurements, to link microbial strains and fermentation-derived metabolites to functional and clinical outcomes. Finally, the development of personalized dietary frameworks should be prioritized. Study designs that stratify participants based on baseline microbiota, dietary background, and host metabolic or immune characteristics will help identify subgroups that are most likely to benefit from yogurt consumption. Together, these approaches will strengthen causal inference and support the translation of yogurt research into more precise and actionable nutritional recommendations.

## 9. Conclusion

Evidence indicates that yogurt may act as a biologically active fermented food capable of influencing gut microbiota composition, microbial metabolism, and host physiological responses. These effects appear to be mediated by coordinated interactions among live microorganisms, fermentation-derived metabolites, and the dairy matrix, leading to changes in microbial diversity, functional outcomes, and host responses. However, the consistency and magnitude of these effects vary across studies due to heterogeneity in yogurt formulations, microbial strains, intervention duration, study design, and individual host factors. Evidence from long-term human studies remains limited, and most mechanistic insights are derived from animal models or short-term interventions. To advance scientific understanding and translational application, researchers should incorporate standardized yogurt formulations, clearly characterized microbial and metabolic profiles, and multi-omics assessments integrated with host phenotypes. Collectively, advancing yogurt research along these lines will strengthen its scientific foundation and enhance its potential role in precision nutrition and gut health promotion.

## Use of AI tools declaration

The authors declare they have not used Artificial Intelligence (AI) tools in the creation of this article.


