# Supplementary material for: Yogurt as a modulator of gut and beyond gut: Mechanisms, health effects, and clinical translation
Source: AIMS Microbiol. 2026 Jun 26;12(2):393–421. doi: 10.3934/microbiol.2026017 (PMC13370263; doi:10.3934/microbiol.2026017)
Supplement: Supplementary file 1 [file microbiol-12-02-017-s001.pdf]

Microbiology

AIMS Microbiology, 12(2): 393–421.
DOI: 10.3934/microbiol.2026017
Received: 01 March 2026
Revised: 08 June 2026
Accepted: 23 June 2026
Published: 26 June 2026

https://www.aimspress.com/journal/microbiology

Review

Yogurt as a modulator of gut and beyond gut: Mechanisms, health effects, and clinical translation

Daiyu Yang[1,2], Hongwen Zhao[3], Kun He[1,4], Wangyang Chen[1,2], Hemiao Xu[1,2], Shuai Li[1,2], Qiming Xiao[1,2], Jinshui Yang[3,*] and Dong Wu[1,4,*]

[1] State Key Laboratory of Complex Severe and Rare Diseases, Department of Gastroenterology, Peking Union Medical College Hospital, Chinese Academy of Medical Sciences and Peking Union Medical College, Beijing 100730, China
[2] Chinese Academy of Medical Sciences and Peking Union Medical College, Beijing 100730, China
[3] State Key Laboratory of Animal Biotech Breeding, College of Biological Sciences, China Agricultural University, Beijing, China
[4] Department of Gastroenterology, The People's Hospital of Xizang Autonomous Region, Lhasa 850000, China

* **Correspondence:** Email: yangjsh1999@163.com; wudong@pumch.cn.

Search strategy

1. PubMed

((yogurt [Title/Abstract]) OR (leben[Title/Abstract]) OR (milk [Title/Abstract]) OR (kefir [Title/Abstract])) AND ((Gastrointestinal Microbiomes [Title/Abstract] OR Microbiome, Gastrointestinal [Title/Abstract] OR Microflora, GI [Title/Abstract] OR GI Microflora [Title/Abstract] OR GI Microfloras [Title/Abstract] OR Microfloras, GI [Title/Abstract] OR Microbiome, GI [Title/Abstract] OR GI Microbiome [Title/Abstract] OR GI Microbiomes [Title/Abstract] OR Microbiomes, GI [Title/Abstract] OR Enteric Microbiota [Title/Abstract] OR Enteric Microbiotas [Title/Abstract] OR Microbiota, Enteric [Title/Abstract] OR Microbiotas, Enteric [Title/Abstract] OR Flora, Enteric Microflora [Title/Abstract] OR Enteric Microflora Flora [Title/Abstract] OR Enteric Microflora Floras [Title/Abstract] OR Floras, Enteric Microflora [Title/Abstract] OR Microflora Flora, Enteric [Title/Abstract] OR Microflora Floras, Enteric [Title/Abstract] OR Gut Microflora [Title/Abstract] OR Microflora, Gut [Title/Abstract] OR Gastrointestinal Microflora [Title/Abstract] OR Microflora, Gastrointestinal [Title/Abstract] OR Gastrointestinal Flora [Title/Abstract] OR Flora, Gastrointestinal [Title/Abstract] OR Gut Flora [Title/Abstract] OR Flora, Gut [Title/Abstract] OR Gastrointestinal Microbial Community [Title/Abstract] OR Gastrointestinal Microbial Communities [Title/Abstract] OR Microbial Community, Gastrointestinal [Title/Abstract] OR Gut Microbiome [Title/Abstract] OR Gut Microbiomes [Title/Abstract] OR Microbiome, Gut [Title/Abstract] OR Gastrointestinal Microbiota [Title/Abstract] OR Gastrointestinal Microbiotas [Title/Abstract] OR Microbiota, Gastrointestinal [Title/Abstract] OR Microflora [Title/Abstract] OR Microfloras [Title/Abstract] OR Gut Microbiota [Title/Abstract] OR Gut Microbiotas [Title/Abstract] OR Microbiota, Gut [Title/Abstract] OR Intestinal Microbiome [Title/Abstract] OR Intestinal Microbiomes [Title/Abstract] OR Microbiome, Intestinal [Title/Abstract] OR Intestinal Microflora [Title/Abstract] OR Microflora, Intestinal [Title/Abstract] OR Intestinal Flora [Title/Abstract] OR Flora, Intestinal [Title/Abstract] OR Intestinal Microbiota [Title/Abstract] OR Intestinal Microbiotas [Title/Abstract] OR Microbiota, Intestinal [Title/Abstract] OR Enteric Bacteria [Title/Abstract] OR Bacteria, Enteric [Title/Abstract] OR Gastric Microbiome [Title/Abstract] OR Gastric Microbiomes [Title/Abstract] OR Microbiome, Gastric [Title/Abstract]) OR ("Gastrointestinal Microbiome" [Mesh]))

2. Web of Science

TS = ((yogurt OR leben OR milk OR kefir) AND (Gastrointestinal Microbiomes OR Microbiome, Gastrointestinal OR Microflora, GI OR GI Microflora OR GI Microfloras OR Microfloras, GI OR Microbiome, GI OR GI Microbiome OR GI Microbiomes OR Microbiomes, GI OR Enteric Microbiota OR Enteric Microbiotas OR Microbiota, Enteric OR Microbiotas, Enteric OR Flora, Enteric Microflora OR Enteric Microflora Flora OR Enteric Microflora Floras OR Floras, Enteric Microflora OR Microflora Flora, Enteric OR Microflora Floras, Enteric OR Gut Microflora OR Microflora, Gut OR Gastrointestinal Microflora OR Microflora, Gastrointestinal OR Gastrointestinal Flora OR Flora, Gastrointestinal OR Gut Flora OR Flora, Gut OR Gastrointestinal Microbial Community OR Gastrointestinal Microbial Communities OR Microbial Community, Gastrointestinal OR Gut Microbiome OR Gut Microbiomes OR Microbiome, Gut OR Gastrointestinal Microbiota OR Gastrointestinal Microbiotas OR Microbiota, Gastrointestinal OR

Microflora OR Microfloras OR Gut Microbiota OR Gut Microbiotas OR Microbiota, Gut OR Intestinal Microbiome OR Intestinal Microbiomes OR Microbiome, Intestinal OR Intestinal Microflora OR Microflora, Intestinal OR Intestinal Flora OR Flora, Intestinal OR Intestinal Microbiota OR Intestinal Microbiotas OR Microbiota, Intestinal OR Enteric Bacteria OR Bacteria, Enteric OR Gastric Microbiome OR Gastric Microbiomes OR Microbiome, Gastric)

AIMS Press

© 2026 the Author(s), licensee AIMS Press. This is an open access article distributed under the terms of the Creative Commons Attribution License (https://creativecommons.org/licenses/by/4.0)
